# The Importance of Telemedicine during COVID-19 Pandemic: A Focus on Diabetic Retinopathy

**DOI:** 10.1155/2020/9036847

**Published:** 2020-10-14

**Authors:** Raffaele Galiero, Pia Clara Pafundi, Riccardo Nevola, Luca Rinaldi, Carlo Acierno, Alfredo Caturano, Teresa Salvatore, Luigi Elio Adinolfi, Ciro Costagliola, Ferdinando Carlo Sasso

**Affiliations:** ^1^Department of Advanced Medical and Surgical Sciences, University of Campania “Luigi Vanvitelli”, Piazza Luigi Miraglia 80138 Naples, Italy; ^2^Department of Precision Medicine, University of Campania “Luigi Vanvitelli”, Via de Crecchio 7, 80138 Naples, Italy; ^3^Department of Medicine & Health Sciences “V. Tiberio”, University of Molise, Via F. De Sanctis, 1, 86100 Campobasso, Italy

## Abstract

Recently, telemedicine has become remarkably important, due to increased deployment and development of digital technologies. National and international guidelines should consider its inclusion in their updates. During the COVID-19 pandemic, mandatory social distancing and the lack of effective treatments has made telemedicine the safest interactive system between patients, both infected and uninfected, and clinicians. A few potential evidence-based scenarios for the application of telemedicine have been hypothesized. In particular, its use in diabetes and complication monitoring has been remarkably increasing, due to the high risk of poor prognosis. New evidence and technological improvements in telemedicine application in diabetic retinopathy (DR) have demonstrated efficacy and usefulness in screening. Moreover, despite an initial increase for devices and training costs, teleophthalmology demonstrated a good cost-to-efficacy ratio; however, no national screening program has yet focused on DR prevention and diagnosis. Lack of data during the COVID-19 pandemic strongly limits the possibility of tracing the real management of the disease, which is only conceivable from past evidence in normal conditions. The pandemic further stressed the importance of remote monitoring. However, the deployment of device and digital application used to increase screening of individuals and monitor progression of retinal disease needs to be easily accessible to general practitioners.

## 1. Introduction

Telemedicine is usually defined as a combination of both technologies and devices able to remotely gain information about a patients' health status, so to aid in deciding if there is a need or urgency to intervene [1]. Hence, it may represent both a screening and diagnostic tool, which demonstrated remarkable importance in recent literature, mostly due to the higher deployment and development of digital technologies (e.g., smartphones and digital connections).

Appropriate tools allow clinicians at reaching and periodically monitoring individuals who have difficulties attending specialist visits, especially patients affected by chronic diseases, who require continuous follow-up. As well as this, on the off chance of a firsthand appointment, therapy could be periodically assessed by sending the data recorded on the digital tool to a specialist.

Two of the major clinical areas covered by telemedicine are cardiovascular diseases and diabetes, alongside all its chronic complications. Particularly, retinopathy, the most widespread diabetes complication, usually needs a fundus oculus examination by an eye-care specialist; however, in rural environments or those who live far from dedicated referral centers, patients either cannot easily attend these examinations or exert a poor adherence to the visit. The latter is mainly due to the disparity between the low number of ophthalmologists and the large population of diabetic patients, as well as to the uncomfortable traditional fundus oculi, which provide pupil dilation after instillation of eye drops.

**Figure 1:**
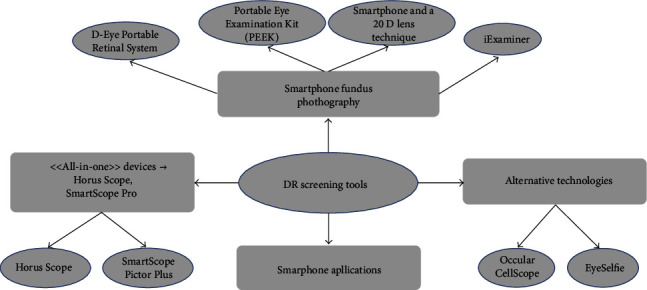
Telemedicine approaches for the screening or the diagnosis of diabetic retinopathy.

Mydriasis has the disadvantage that patients must wait both for performing the exam and to come back to the daily routine. Conversely, telemedicine can counterbalance this burden, as suggested by current guidelines. In fact, after appropriate training, all clinicians and dedicated clinical personnel can easily take photos of the fundus oculi with nonmydriatic fundus cameras (Figure 1) [2]. Retinal digital photos, though not representing the gold standard, may be equally used to screen the diabetic population and differentiate among the different stages of diabetic retinopathy (DR), thus referring to the ophthalmologist only ungradable images or suspicious cases.

Herein, we analyze the state of the art of telemedicine during the pandemic, particularly focusing on the management of diabetes and its complications. In fact, during the COVID-19 pandemic, due to the mandatory social distancing imposed to prevent the outspread of infection, the use of telemedicine in diabetes monitoring has been remarkably increasing.

## 2. The Importance of Telemedicine in a Pandemic

As aforementioned, telemedicine has initially arisen to provide medical assistance either in rural areas or where access to care is hard, mainly aimed at improving chronic disease management [3], mostly in urgencies [4]. Over the years, the onset of either epidemics or pandemics has led to the employment of increasingly novel digital technology strategies, which have also triggered the use of telemedicine during the diverse stages of the infection much more frequently, such as in the cases of the SARS epidemic in 2003 and, later, MERS‐CoV in 2013 [5].

Due to its novelty, as well as the large spectrum of potential applications, a clear differentiation of settings in which to use telemedicine during emergency periods has also been challenging. A few potential evidence-based scenarios have been hypothesized in 2015 [6]. For example, e-health can be applied to all asymptomatic subjects in an epidemic area. This “home-based” management is most useful in the suspect of infection-related symptoms and allows to address subjects to dedicated referral centers. Moreover, positive asymptomatic subjects can be followed up by periodic phone and web consulting. Beyond these, over the last years, digital geolocalization tools further contributed to an improvement of these services. In addition, telemedicine is also useful to take care of individuals either in domiciliary or nosocomial isolation. In this latter case, telemedicine ensures an adequate safety to both clinicians and caregivers limiting the direct contact with the infected patients only to strictly nondeferrable urgencies [7]. Lastly, up to now, telemedicine can also support the outpatients' management of periodical visits, which are halted due to the mandatory lockdown imposed by local governments.

Among chronic diseases, cardiovascular diseases particularly require constant monitoring, thus increasing the risk of infection both for patients and clinicians [8]. In this setting, remote monitoring has been expanding also beyond emergency situations, to observe a rapid enhancement of e-health technologies during either epidemics or pandemics. As an example, electrophysiologists have been converting most clinical visits to remote monitoring (phone, video calls for visits, review of data from digital wearables, etc.) [9, 10], as well as cardiac implantable electronic device checks, whenever feasible [11], as suggested as class I recommendation [12]. Where possible, nonurgent procedures should be postponed or, in the case of need, coordinated on the same day of visit to minimize multiple exposures, whilst postprocedural follow-up should be performed remotely.

In this sense, the pandemic caused by SARS-CoV-2 coronavirus has been giving a remarkable impulse of the management of other chronic diseases.

### 2.1. Focus on COVID-19 Evidence

The World Health Organization (WHO), after the large expansion of SARS-CoV-2 virus, declared the state of pandemic by coronavirus 2019 disease (COVID-19) on March 11, 2020. The COVID-19 outbreak has triggered the lockdown of populations worldwide, strongly affecting daily life, as well as most health systems, which have been faced with the management of both infected patients and routine non-COVID-19 patient care.

Thanks to both positive evidence from previous epidemics/pandemics and technological advancements, during this critical period, the use of telemedicine has increased, especially in industrialized countries, mostly the United States [13], the United Kingdom, and China [14, 15].

Much more novel digital technologies have supplemented common phone interviews. Both national and international guidelines should consider the introduction of e-health technology in their updates, strictly differentiating recommendations for common use and emergency situations.

As a matter of fact, the lack of either vaccines or effective treatments, due to social boundary and lockdown as main preventive measures, renders telemedicine the safer interaction system between patients and clinicians [16].

The large proportions of this pandemic have also encouraged a reduction of the gap related to the poor compliance to the use of digital tools.

Based on past evidence and previous models, during the COVID-19 pandemic, social distancing has triggered three potential e-health applications [6, 17, 18]. On the one hand, patients at higher risk of infection, especially those with either chronic, autoimmune, or immunosuppressant diseases can prevent the exposure to risk factors by virtually communicating with their general practitioner and/or specialist. Referral to clinical facilities is thus limited to extreme needs.

In addition, novel strategies of telephonic triage have been proposed, which allows for better screening of suspect SARS-CoV-2 cases, reducing referral to firsthand aid of a huge amount of people worried about potential infection by SARS-CoV-2. Definite positive cases, either asymptomatic or mildly symptomatic, do not obtain the priority to hospitalization; nonetheless, they are carefully followed up by dedicated channels with both COVID-19 centers, general practitioners, and local health authorities. Further, e-health communication has helped mildly infected clinicians not to discontinue their routine practice [19, 20], providing them with the opportunity to pursue their activities remotely. The feasibility of this innovative medical approach is still the object of debate [21]. Moreover, the cost-to-benefit ratio of these tools should be maximized for a better global utilization of telemedicine in the next future beyond the current emergency setting, with a higher focus on chronic disease management [22, 23].

## 3. Diabetic Retinopathy Management during COVID-19

Up to now, several studies worldwide have evaluated the efficacy of telemedicine in different clinical settings, especially focusing on a very specific medical branch, ophthalmology. In fact, teleophthalmology has been assessed in numerous clinical subsettings. As an example, several studies have considered telemedicine usefulness in glaucoma diagnosis, with findings mostly comparable. The largest study, conducted in the UK on over 24,000 subjects, showed an agreement of 87% between optometrist and ophthalmologist examinations (Cohe's kappa = 0.69) [24]. Similar findings were obtained in Kenya, where teleglaucoma disclosed a sensitivity of 41.3% and a specificity of 89.6% as compared to the standard fundus oculus exam by the eye-care specialist [25]. A similar study was led in Canada on innovative smartphone applications, even though, despite the similar findings, images were ungradable in 24% of cases [25]. However, smartphone ophthalmoscopy showed substantial agreement with slit-lamp examination for the estimation of the vertical cup-to-disc ratios in glaucoma screening [26].

Evidence on e-health technologies on cataract and age-related macular degeneration screening is still poor and on small populations, even the modest literature on these topics has shown an overall good quality of acquired images in 93-100% of cases [27].

As briefly mentioned before, among chronic diseases, diabetes, alongside its diverse complications, renders patients at high risk of poor prognosis [28, 29]. Over the years, telemedicine has been proven as a useful tool to allow for periodic management of glycemic levels [30]. Telemedicine has been further implemented to gain an effective screening of complications without requiring mandatory on-site visits. Lack of time, distance from specialized centers, disabilities, and long waiting lists are among the most common causes of a limited access of patients to specialty visits. In the case of pandemics, social distancing further contributes [31–35].

Of interest, the development of digital devices for glycemia monitoring (e.g., glycemic holters and micropump) both in type 1 and type 2 diabetic patients has allowed the deployment of an easier self-monitoring of glycemia. In fact, data are rapidly collected and transferred either to the specialist or the general practitioner (e.g., by email or digital systems), who could consider eventual therapy modifications, as well as deeper diagnostic/therapeutic urgent investigations [36–38]. Many studies have also demonstrated an increased rate of HbA_1c_ target achievement among users of glycemic monitoring devices rather than controls, with a subsequent reduction also in the risk of complications [39–42].

Telehealth may also help in the screening of diabetes complications. In particular, for years, diabetic retinopathy has been the object of screening and monitoring programs. Fundus cameras and other portable devices make it possible to take retinal photos, which can be sent to specialized referral centers for reading, by both clinicians and technicians. Available data worldwide have demonstrated the efficacy and usefulness of telemedicine in this context. Thanks to these tools, screening has been extended to a much larger portion of diabetic subjects, and the comparison between telemedicine and standard fundus oculus exam has revealed a good efficacy from the use of nonmydriatic cameras both in terms of sensitivity and specificity [43].

In India, a recent screening investigation by Fundus on Phone (Remidio FOP), a smartphone-based imaging device, allowed for DR diagnosis in over 3,500 patients (22.8% of the entire study population) [44, 45]. As well as this, after a 6- and 18-month follow-up, telemedicine has been reported to significantly increase the individuals screened for DR [46]. A further study, on about 100 diabetic patients, reported a sensitivity of 97.1% and a specificity of 95% of fundus oculus photos in detecting mild nonproliferative DR (NPDR) than the standard mydriatic exam. In moderate NPDR assessment, though demonstrating with a similar specificity (96.9%), sensitivity was lower (53.3%) [47]. Conversely, another study on a similar sample size showed both a high sensitivity (85%) and specificity (90%) in the diagnosis of moderate NPDR with 2-field 50-degree nonstereo digital fundus photographs than slit-lamp ophthalmoscopy performed by an ophthalmologist [48]. A larger scale teleretinal screening was deployed on a sample of over 20,000 people from the US diabetic population [49].

In our experience of the NO BLIND study, a multicenter cross-sectional study, almost 1,500 diabetic subjects were screened for DR by a digital smart ophthalmoscope. Fundus oculus photos performed by trained diabetologists were diagnostic for diabetic retinopathy in 15.5% of the study population, with both a high sensitivity and specificity (94.3% and 100%, respectively), as compared with standard fundus oculus examination in mydriasis [50].

In the last years, at the same time, many smartphone applications have been developed to acquire nonmydriatic images of any area of the eye [51]. Some recent studies, consistent with our estimates [50], have predicted a potential increase of screened patients of 50-60% [52–54], also thanks to a remarkable improvement of image quality [53, 55, 56]. Similarly, two screening programs conducted both in Africa and Canada were aimed at improving both health and life quality of people affected by diverse retinal diseases, as well as at reducing complication frequency [57, 58].

Moreover, teleophthalmology disclosed a good cost-to-efficacy ratio, both in terms of human and monetary resources, and travelling costs for patients and public health systems [59]. It has been recently demonstrated, in a meta-analysis focused on cost efficacy assessment of ophthalmologic screening programs by telemedicine in several countries (e.g., the United States, Canada, Singapore, India, Brazil, and South Africa), that, though with an initial increase of costs related to devices and training, over time, there is an economic saving [31]. Likewise, in our multicenter experience, remarkable monetary savings from the use of retinal cameras rather than traditional exam (estimated mean cost per patient equal to €3.02 vs. €7.75) were observed, as well as time savings both for patients and clinicians (about 2/3 minutes vs. 20/30 minutes) [50].

Even though several studies support diabetic retinopathy screening by digital technology (Table 1), up to now, no national screening program has aimed at DR prevention and diagnosis.

Evidence about diabetes remote monitoring during the COVID-19 pandemic is still poor. Recent diffusion of the pandemic and lack of data strongly limit the possibility of tracing the real management of the disease. We may only hypothesize, from previous data on subjects already using these devices, that data transmission allows for distant monitoring. A similar approach can be considered for diabetic retinopathy; hence, it is reasonable that general practitioners adopt either retinal cameras or other devices to promote an effective control also during pandemics.

## 4. Conclusions

Current emergency status due to the pandemic could represent a further stimulus to the diffusion of large telemedicine screening programs in routine clinical practice. New evidence and technological improvement of devices have made telemedicine a useful solution for diabetic retinopathy screening. These advancements could be useful to widen the number of individuals screened and monitor progression of retinal disease, both in conditions of pandemics/urgencies and in routine clinical practice. The Campania region was among the first and few Italian regions to legislate in favor of telemedicine. Writing the decree signed by the General Director of the Regional Health Service (protocol 2020_0175167 on 27/03/2020), telemedicine was approved and regulated as a tool for diabetes treatment. The document describes the methods and the paths to follow, the actors, and the users. Therefore, diabetologists are invited to register and report all the procedures and methods carried out. In this way, once the COVID-19 pandemic is over, it will be possible to analyze approaches and results in order to transform telemedicine as a commonly used tool. This is the challenge that diabetologists are pursuing, especially in the field of diabetic retinopathy.

However, the deployment of such devices and digital applications needs to be rendered easier and accessible without specific long training. Additionally, the cost-to-benefit gap should be minimized.

Thus, it would be hopeful to extend the use of telehealth systems to general practitioners, as well as to increase the proportion of users among patients themselves. As already observed in other microangiopathic complications of diabetes [60], for the concerns raised by DR, an integrated assessment of both first- and second-class medical care would be desirable. The majority of patients could keep remotely connected with their specialist, overcoming common barriers, especially in definite areas, and still contain the spread of the virus by social distancing.

## Figures and Tables

**Table 1:** Telemedicine approaches for the screening or the diagnosis of diabetic retinopathy (DR).

	Country	Sample size	Approach	Sensitivity/specificity	Usefulness	Savings
Bawankar et al. [43]	India	560	Bosch nonmydriatic fundus camera	Sensitivity: 91.2%, specificity: 96.9%	Large compliance and accessibility to medical care in rural areas	Estimated decreased costs
Rajalakshmi et al. [45]	India	301	Carl Zeiss fundus camera and “Fundus on Phone” (FOP)	Sensitivity: 92.7%, specificity: 98.4% (for STDR ➔ 87.9% and 94.9%)	Sleekness, easy portability, and wireless connectivity ➔ easily usable in nonhospital settings	Use of long-life LED illumination and lithium-ion battery in FOP reduces the operational cost of FOP
Liesenfeld et al. [48]	Germany	129	Slit-lamp biomicroscopy	Median sensitivity = 85%, median specificity = 90%	New perspectives ➔ send images for instant review by a retinal expert	Estimated decreased costs
Sasso et al. [50]	Italy	1461	Horus Scope	Specificity = 100%, sensitivity = 94.3%	Eye-care services available to everyone at a sustainable cost	Estimated mean cost per patient €3.02 vs. €7.75 of traditional fundus oculus examination
Russo et al. [53]	Italy	120	Smartphone ophthalmoscopy (D-Eye)	NPDR—specificity: 95%, sensitivity: 80% PDR—specificity: 100%, sensitivity: 89%	Portability, affordability, and connectivity of a smartphone ophthalmoscope	Relatively low hardware and production costs (final retail price <$300)
Andonegui et al. [55]	Spain	1223	Nonmydriatic retinal camera (TRC NW6S, Topcon, USA)	Specificity: 83%, sensitivity: 91%.	Cheaper, less time-consuming, easily applicable to populations far from the specialists, no requirement of pupil dilation	More cost-effective than traditional methods of DR screening
Gomez-Ulla et al. [59]	Spain	—	Nonmydriatic fundus camera (Canon, Model CR5-45NM)	—	Larger accessibility	Reduced costs Digital image ➔ €5.31/pt (at no cost at the endocrinologist consultation) Direct examination ➔ €3.13 + €48.29 (cost of ophthalmologist attendance)
